# GIS-modelled built-environment exposures reflecting daily mobility for applications in child health research

**DOI:** 10.1186/s12942-020-00208-2

**Published:** 2020-04-10

**Authors:** Amy Mizen, Richard Fry, Sarah Rodgers

**Affiliations:** 1 Health Data Research UK (HDR-UK), Data Science Building, Swansea University, Swansea, SA2 8PP UK; 2 National Centre for Population Health and Wellbeing Research, Swansea University Medical School, Swansea, SA2 8PP UK; 3 Institute of Population Health Sciences, University of Liverpool, Liverpool, L69 3BX UK

**Keywords:** Environmental exposure, Child health, Walking, Daily mobility, School commute, Weighted network

## Abstract

**Background:**

Inaccurately modelled environmental exposures may have important implications for evidence-based policy targeting health promoting or hazardous facilities. Travel routes modelled using GIS generally use shortest network distances or Euclidean buffers to represent journeys with corresponding built-environment exposures calculated along these routes. These methods, however, are an unreliable proxy for calculating child built-environment exposures as child route choice is more complex than shortest network routes.

**Methods:**

We hypothesised that a GIS model informed by characteristics of the built-environment known to influence child route choice could be developed to more accurately model exposures. Using GPS-derived walking commutes to and from school we used logistic regression models to highlight built-environment features important in child route choice (e.g. road type, traffic light count). We then recalculated walking commute routes using a weighted network to incorporate built-environment features. Multilevel regression analyses were used to validate exposure predictions to the retail food environment along the different routing methods.

**Results:**

Children chose routes with more traffic lights and residential roads compared to the modelled shortest network routes. Compared to standard shortest network routes, the GPS-informed weighted network enabled GIS-based walking commutes to be derived with more than three times greater accuracy (38%) for the route to school and more than 12 times greater accuracy (92%) for the route home.

**Conclusions:**

This research advocates using weighted GIS networks to accurately reflect child walking journeys to school. The improved accuracy in route modelling has in turn improved estimates of children’s exposures to potentially hazardous features in the environment. Further research is needed to explore if the built-environment features are important internationally. Route and corresponding exposure estimates can be scaled to the population level which will contribute to a better understanding of built-environment exposures on child health and contribute to mobility-based child health policy.

## Background

Understanding how exposure to the built-environment impacts on human health has received increased attention over the past two decades. Public health issues such as obesity, diabetes and common mental health disorders are not being affected by current policies and interventions with global trends showing an increase in these non-communicable diseases. It is imperative for researchers, policy makers and practitioners to better understand the role of the built-environment on health. A thorough understanding of how health-outcomes are associated with the built-environment provides evidence for action to improve physical and mental health for whole populations.

It is well established that daily mobility is a key determinant of built-environment exposure as it defines when, where and how people are exposed to different physical and social environments. Daily mobility describes the spatiotemporal patterns of an individual’s movement during their day-to-day life [1, 2]; including three key factors: spatial, temporal and the nature of activities [1].

Daily mobility has been measured subjectively using retrospective surveys such as life-space assessment [3], travel diaries [4] and interactive map-based questionnaires [5]. Alternative methods have used Geographic Information Systems (GIS) to model daily mobility, but these methods often over simplify spatiotemporal patterns of an individual’s everyday movement in their environment. Particularly for children where route choice is complex and social factors influence where a child may travel [6]. Studies using GIS-modelled exposures often focus on a single aspect of daily mobility (e.g. home or school/work environment), however, it is unlikely for an individual to only be exposed to one environment in day-to-day life. Individuals visit multiple locations at different times of day and are therefore exposed to numerous environments (e.g. home, school/work, hobbies). It is therefore important to capture multiple exposure environments when looking at the influence of the built environment on health.

Commuting is one aspect of daily mobility commonly represented by Euclidean distances or shortest network routes. However, these measures have been found to be insufficient in representing aspects of child daily mobility such as commuting routes, particularly children who walk to and from school [7–9]. Using the shortest network route (SNR) as a proxy for the route a child takes to school has been found to be a reliable proxy for the distance they travel, but not for the environment they are exposed to. This is especially true for children who have a walking commute to and from school, where route choice is often more complex than just the shortest route. Furthermore, the route taken to school can differ to the route home from school [8]. Characteristics of the built-environment, such as road type, woodland and traffic lights, have been found to be associated with route choice [10–12]; in particular the walk home from school. Studies using GIS-modelled exposures have not differentiated between the route to school and route home in their modelling.

A preferred method to measure exposure in children is to use GPS recording devices as these instruments have become a reliable and accurate way to objectively measure individual-level daily mobility [13–15]. Studies have investigated adult and child daily mobility and corresponding exposures using GPS data for a range of built-environment exposures such as fast food outlets, greenspace and pollution. Many of these studies that have explored children’s daily mobility [16, 17] have focussed on the importance of the school commute; investigating physical activity levels and exposure to the ‘retail food environment’ (RFE) along active commutes [8, 10, 18–21]. However, daily mobility measures derived from GPS data can be expensive and time consuming to implement and are therefore not practical for recording and analysing population-level exposures [22]. Population-level research is essential to explore and understand similarities and differences across and within populations. To bring about large-scale changes in a population’s health, policy needs to be based on evidence from national-scale studies. Evidence has shown that even small improvements in health at an individual-level can lead to a substantial gain at a population-level [23].

To address limitations in previous studies, this paper presents a method that can be used to model population-level, daily commuting routes for children that walk to school. The method contributes to improving individual-level daily mobility measures and thus measuring exposure environments for children through four steps:

i. We processed GPS data for a cohort of 995 children aged 13–14 years and identified walking routes *to school* and *home*. We generated SNR from home to school for children who walked *to school* and/or *home*.
ii. We calculated built-environment characteristics along the GPS and SNR routes. We used logistic regression analyses to identify the most important built-environment characteristics along routes *to school* and *home*. This meant we could weight roads based on objectively collected data to model child preference for route choice.
iii. We generated routes *to school* and *home* using a weighted network. We used odds ratios from the logistic regression analysis to inform cost values on a network. We used this weighted network to produce predicted weighted network routes (WNR) *to school* and *home*.
iv. We validate our predictions by comparing the WNR with GPS route data using intersection analyses. We also calculate exposure to the RFE and undertake multilevel regression analyses to validate exposures generated from our modelled routes.

This research contributes to childhood built-environment exposure research that involves spatial mobility assessment, as it highlights the potential to generate population-level exposures using GIS. Moreover, enhanced knowledge of modelling the daily exposure to the built-environment at a population-level can be applied to different subgroups of the population and numerous exposures such as fast food, physical activity opportunities, pollution and greenspace.

## Methods

All GIS and GPS data processing and analysis was undertaken in a PostGIS database [24] using pgadmin3 version 9.5 [25]. GIS-generated routes were calculated using pgRouting [26], a geospatial routing extension for PostGIS databases. All statistical analyses were undertaken using R version 3.3.3.

### GPS data processing and computation of routes to school

The GPS data was provided by researchers who had worked on a large-scale study with 995 children aged 13–15 [27]. Data collection methods are reported in detail elsewhere. In summary, the Physical Environment and Activity Relationships (in adolescents) (PEAR) study was a cross-sectional study of students from 15 schools across Bristol, South Gloucestershire, North Somerset, and Bath and North East Somerset aged 13–14 years old. A University Ethics Committee approved the study written informed consent was obtained from a parent or guardian of all participating adolescents. Data were collected between November 2012 and March 2014 [28].

#### GPS data processing

**Fig. 1:**
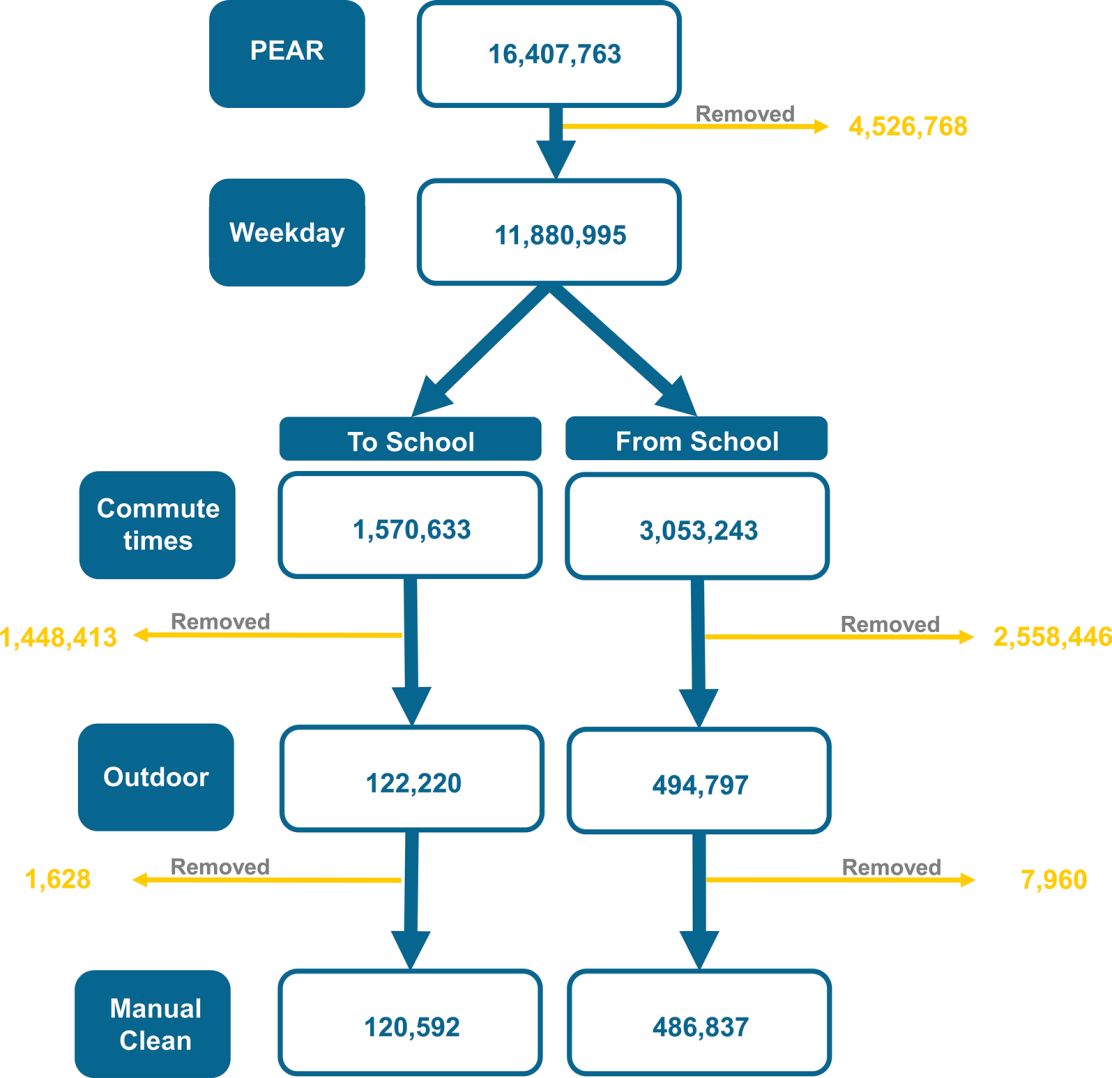
Workflow of GPS point data processing (n)

Raw GPS data was received by the data providers. For each PEAR participant, home locations as XY coordinates and the name of the school attended was provided. Home and school building footprints were extracted from OS Mastermap Topography Layer [29], provided by DigiMap [30]. All data were imported into a PostGIS database using the command line tool pgfutter [31]. A criteria-based approach was used to prepare the GPS data for analysis. The method was based on published criteria-based methods [32, 33] and comprised of three main stages: pre-processing; processing and post-processing. This workflow is summarised in Fig. 1.

##### Pre-processing GPS data (inclusion criteria)

Inclusion criteria were developed to select points exclusively from the route *to school* or *home from school*. Individuals were excluded that did not contain both home and school location (n = 5). The inclusion criteria were:

i. Points recorded on a weekday.
ii. Points recorded between (07:30 and 09:30) and (14:30: and 16:30).
iii. Points recorded outside school building footprint.
iv. Points recorded more than 40 m away from home location.
v. Points recorded outside of any building footprints.

##### Processing GPS data

The GPS points were indexed to categorise individual trips made by each participant. For each trip, GPS points were ordered by their timestamp. A trip was defined as consecutive points on a single day during morning commute time (between 7:30 and 9:30), or during afternoon commute time (between 14:30 and 16:30). Each trip was assumed to represent a participant travelling from home to school, or from school to home. The GPS points for each trip were then aggregated to create line geometries. Home and school locations were appended on to each end of the line geometries because they had been removed in the pre-processing stage. Walking routes were extracted from the data based on average speed of the points that made up each trip (average speed < 10 m/s) [32].

##### Post-processing

Remaining outliers were removed by selecting points that were more than 100 m from any other GPS points belonging to that trip. Routes were manually inspected to ensure that only the route from home *to school* and *home* had been captured. Any points representing other journeys were removed e.g. after some children arrived home, they left home again before 16:30. These points were removed manually.

#### Modelled routes to school and home from school

SNR from home to school and school to home were calculated for each PEAR participant that walked to school and/or home from school. The SNR were generated using centrelines from an open source road network downloaded from OpenStreetMap [34].

### Built-environment characteristics

#### Generating built-environment characteristics

Characteristics of the built-environment known to influence child route-choice were calculated for GPS walking routes and the corresponding GIS-generated SNRs. Table 1 defines the built-environment characteristics that were calculated, how they were calculated and underlying data sources.

**Table 1:** Built-environment characteristics calculated along GPS and SNR routes in PostGIS; including how built-environment characteristic was defined and data source

Built-environment characteristic	Definition	Data source
Length of route (km)	Length of route in kilometres	Line geometries were downloaded from OSM [34]
Bluespace (%)	Percentage of route along visible water (e.g. river, canal)	Bluespace polygon data were downloaded from OSM [34]
Traffic lights (n)	Total number of traffic light signals along route	Traffic light point data obtained from OSM [34]
Accidents (n)	Total number of police-recorded traffic-related incidents along route	Road traffic accident data was downloaded from Stats19 [35]. Accidents that occurred between the school commuting hours (7:30–9:30 and 14:30–16:30) were extracted and represented as point data
Type of street (%) a. Main road b. Residential c. Minor Road d. Footpath	Percentage of route along this road type	The OSM road types were aggregated into four road classifications that have been used in the literature [11]: main road, minor road, residential road and footpath
Woodland	Percentage of route that has woodland within 25 m of the route	Woodland polygon data were downloaded from OS Meridian 2 [36]
Food outlets	Total number of food outlets within 100 m of route	Postcode level food outlet point data were downloaded from the Food Standards Agency [37]

#### Statistical analyses

The built-environment characteristics listed in Table 1 were included as independent variables in conditional logistic regression models to discriminate between the built-environment characteristics along the GPS routes and the SNR. We fitted two models, A and B for the routes, *to school* and *home* respectively. A backward stepwise method was used to fit the models:

$$P\left( Y \right) = \frac{1}{{1 + e^{{ - \left( {b_{0} + b_{1} X_{1i} + b_{2} X_{2i} + \cdots b_{n} X_{ni} } \right)}} }}$$

where P(Y) is the probability of Y occurring, e is the base of natural logarithms and b_0_ is the constant. For j = 1,…,n X_ji_ are the predictor variables and b_j_ are the predictor coefficients.

### Generating routes to school and home using a weighted network

#### Building a weighted network

**Fig. 2:**
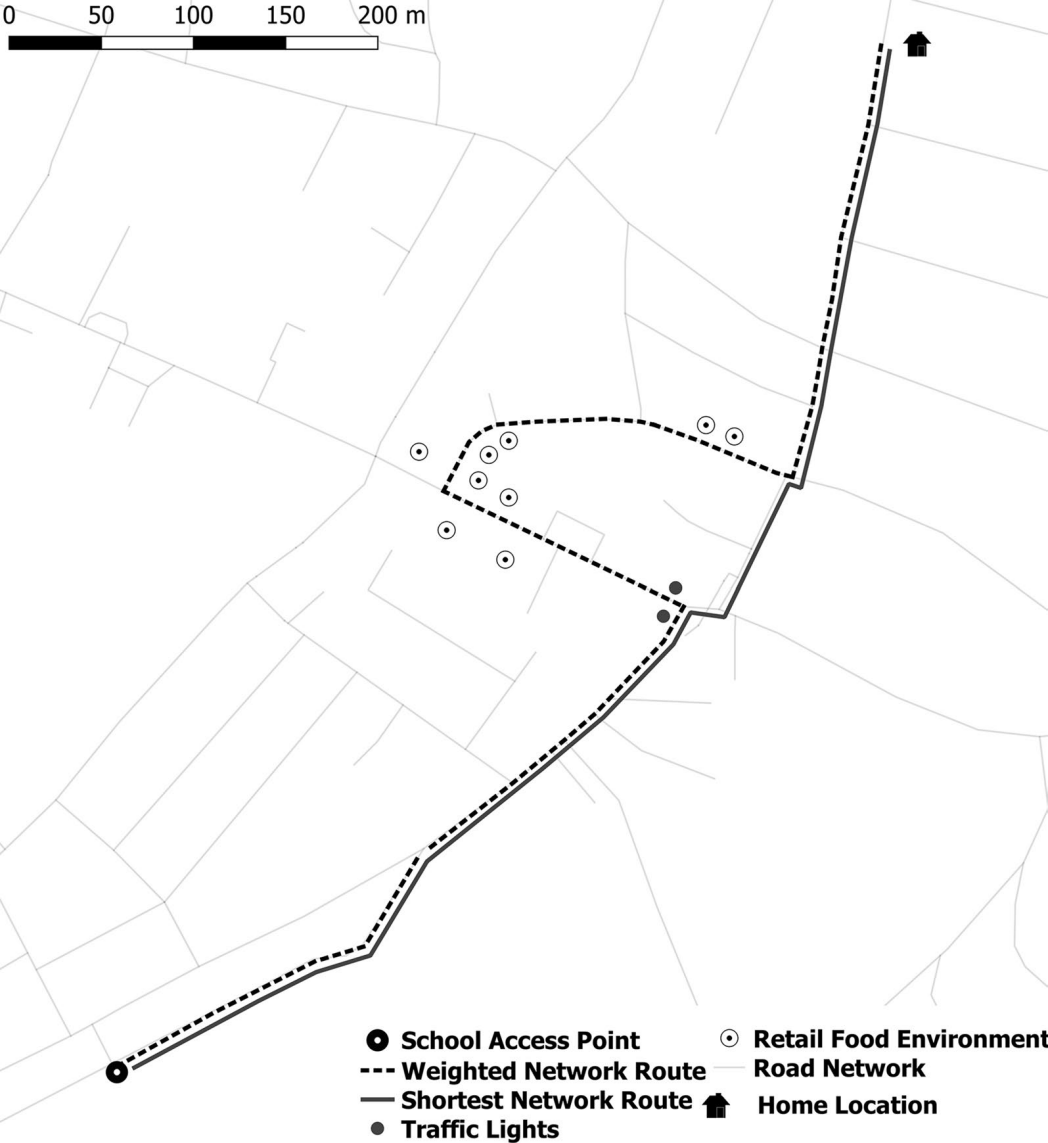
Representation of the impact of cost values on modelled routes

Centrelines downloaded from OpenStreetMap [34] were the basis of the road network. Odds ratios derived from the logistic regression models to discriminate between the GPS route and SNR were used to inform cost values that were assigned to network arcs. Cost-values were applied to every network arc based on road type (Table 6). Then, network arcs were flagged where traffic lights (point geometry) and food outlets (point geometry) were within 25 m of the network arc. If the arc was within 25 m of a food outlet, the arc was reassigned an impedance cost (Fig. 2).

Initially, where there were more built-environment characteristics on GPS routes compared with SNR (e.g. traffic lights had an OR > 1), a lower cost impedance was assigned to on the network. Network arcs that contained built-environment characteristics that were fewer along GPS routes compared to SNR were allocated a higher impedance cost on the network (e.g. main roads had an OR < 1).

A simple machine learning approach was then implemented to find the optimum impedance costs to improve the accuracy of GIS generated routes to school and home from school compared with GPS routes. The final impedance costs are reported as the optimal weights to apply when generating cohort-level GPS walking routes to school and home from school.

#### Generating routes using the weighted network: weighted network routes (WNR)

The Dijkstra algorithm [38] was used to calculate a single route to school and home with minimum cost in terms of distance travelled for each person. The WNR *to school* and *home* were then used to calculate associated exposures to the RFE along the commute to and from school. The weighted network exposures (WNE) to the RFE were defined as the number of food outlets along the WNR within 100 m of the route [8, 10, 15, 39]. We defined the RFE using postcode level, food outlet point data downloaded from the food standards agency [40] to represent trading outlets that were open when the GPS routes were collected.

#### Statistical validation of WNR

Similarity between GPS route shape and WNR shape was determined by calculating the percentage of WNR that intersected within 50 m of the corresponding GPS routes [8]. Descriptive statistics of the intersection between GPS routes and WNRs were calculated.

A multilevel linear regression model was fitted to assess the association between exposure to the RFE along GPS routes and WNR. Multilevel linear regression analyses were fitted to account for the hierarchical structure of the data; route exposures for individuals, and individuals were clustered within 15 schools. Separate models were fitted to assess the relationship between GPS exposures and WNE to the RFE for routes *to school* routes *home from school*. All statistical analysis was undertaken in RStudio (version 3.3.3).

## Results

### GPS routes and route characteristics

In total, 463 individuals provided GPS data. Specifically, 333 individuals provided GPS data for both their walking route to school and walking route home, 44 individuals provided GPS data exclusively for their walking route to school, and 86 individuals provided GPS data only for their walking route home from school. The GPS data yielded 949 walking routes *to school* for 377 individuals and 976 walking routes *home from school*, were recorded for 409 individuals. Tables 2 and 3 summarise the built-environment characteristics of the GPS walking routes recorded in the GPS dataset. As described above, some individuals provided data for their walk *to school* and others for the route *home from school*. The results generally characterise walking routes to school and walking routes home from school. The mean distance recorded by the GPS data for walking routes to school was 1.5 km. The shortest route was recorded at 0.7 km and the maximum distance travelled walking to school was 5.2 km. The median values of the routes to school were, 20% of the route was along main roads, 52% along residential roads and 23% along footpaths. The median number of food outlets on the walk to school was 3. The mean distance travelled on the route home was 1.5 km. The minimum distance walked on the route home was 0.1 km and the maximum distance walked was 7.2 km. The median percentage of road type traversed did not differ greatly from morning routes for main roads, residential roads and footpaths (20%, 51% and 23% respectively). The percentage of the route along footpaths was greater for routes *home* compared with routes to school. The median number of food outlets on the walk *home* was 2 outlets. The range of route length for routes home was greater than routes to school by 1.9 km.

**Table 2:** Summarises the environmental characteristics along the walking routes *to school* recorded in the PEAR dataset

	Mean	Median	Min	Max	Range	Skew	Kurtosis	SD
Distance (km)	1.55	1.41	66	5.18	5.12	0.79	0.68	0.88
Bluespace (%)	2	0	0	43	43	4.44	23.83	5.12
Traffic lights (n)	0	0	0	12	12	4.04	20.02	1.25
Pedestrian crossing (n)	1	0	0	9	9	2.69	7.57	1.48
Accidents (n)	2	1	0	21	21	2.04	4.24	3.39
Main road (%)	27	20	0	100	100	0.72	− 0.67	26.33
Residential road (%)	55	52	0	100	100	0.11	− 0.98	25.33
Footpath (%)	28	23	0	94	94	0.83	0.01	22.13
Minor road (%)	11	5	0	97	97	2.32	6.40	15.77
Food outlets (n)	6	3	0	70	70	3.00	10.80	9.44

Bluespace (%) describes the percentage of the route that is within 50 m of and bluespaces. Main road, residential road, footpath and minor road describe the percentage of the route that traverses these road types. Traffic lights, Pedestrian crossing, accidents and exposure are counts of these features

**Table 3:** Summarises the environmental characteristics along the walking routes home recorded in the PEAR dataset

	Mean	Median	Min	Max	Range	Skew	Kurtosis	SD
Distance (km)	1.51	1.37	0.10	7.17	7.07	1.15	2.36	0.913
Bluespace (%)	2	0	0	39	39	4.11	20.73	4.97
Traffic lights (n)	0	0	0	13	13	4.78	29.96	1.16
Pedestrian crossing (n)	1	0	0	10	10	2.66	8.00	1.36
Accidents (n)	2	1	0	24	24	2.70	8.98	3.36
Main road (%)	26	20	0	100	100	0.76	− 0.48	25.81
Residential road (%)	53	51	0	100	100	0.21	− 0.96	25.77
Footpath (%)	29	23	0	98	98	0.77	− 0.24	24.09
Minor road (%)	11	6	0	98	98	2.46	7.23	15.91
Food outlets (n)	5	2	0	63	63	3.30	13.71	8.38

Bluespace (%) describes the percentage of the route that is within 50 m of bluespaces. Main road, residential road, footpath and minor road describe the percentage of the route that traverses these road types. Traffic lights, pedestrian crossing, accidents and exposure are counts of these features

### Built-environment characteristics along routes to and from school

Table 4 shows the results of the conditional logistic regression for walking routes *to school*. Table 5 shows the results of the conditional logistic regression for walking routes *home*.

**Table 4:** Conditional logistic regression results for environmental characteristics along walking routes *to school* (reference group is shortest network routes)

	Odds ratio	95% CI for OR	
		Lower	Upper
Length (m)	1.00	1.00	1.00
Blue space (%)	1.15	1.07	1.24
Traffic light (n)	1.46	1.11	1.92
Main road (%)	0.91	0.89	0.93
Residential road (%)	0.88	0.86	0.90
Footpath (%)	0.87	0.85	0.89
Minor road (%)	0.91	0.88	0.93
Food outlets (n)	0.92	0.83	0.98

**Table 5:** Conditional logistic regression results for environmental characteristics along walking routes *home* (reference group is shortest network routes)

	Odds ratio	95% CI for OR	
		Lower	Upper
Length (m)	1.00	1.00	1.00
Blue space (%)	1.09	1.03	1.17
Traffic light (n)	1.77	1.33	2.37
Main road (%)	0.92	0.90	0.94
Residential road (%)	0.90	0.87	0.91
Footpath (%)	0.89	0.87	0.91
Minor road (%)	0.91	0.88	0.94
Food outlets (n)	0.90	0.84	0.96

#### Route to school

The odds ratios (OR) in for routes *to school* indicated that along the route *to school*, 15% (OR 1.15, 95% CI 1.07, 1.14) more of the GPS route was along a blue space and contained 46% (OR 1.46, 95% CI 1.11, 1.92) more traffic lights compared to SNR. The ORs for main roads, residential, footpaths and minor roads indicate that the GPS routes contained a significantly smaller percentage of the route along these road types. The GPS routes had a mean number of food outlets of 6 and the OR suggested this was 8% less than exposure along the SNR for the walk *to school* (OR 0.92, 95% CI 0.83, 0.98).

#### Route home from school

The odds ratios (OR) for routes *home from school* indicated that there were 9% (OR 1.09, 95% CI 1.03, 1.17) more blue spaces along the GPS routes compared to SNR and 77% (OR 1.77, 95% CI 1.33, 2.37) more traffic lights along the walk *home from school*. The percentage of the routes along main roads, residential, footpaths and minor roads was significantly less for GPS routes compared with the SNR. The GPS walking routes *home* had a mean value of 5 outlets which was 10% less than the SNR (OR 0.90, 95% CI 0.84, 0.96).

### Weighted network routes to school and home from school

#### Weighted network

Table 6 reports the cost values that were assigned to the road network. Cost values were informed by the OR of the conditional logistic. Network vertices that had traffic lights or outlets along them were reassigned the impedance value set for traffic lights and outlets, as road type impedance values were assigned first. For example, a vertex representing a main road that contained traffic lights was reassigned a value of 0.5. If the vertex was within 25 m of a food outlet, the vertex was reassigned a value of 0.8. Figure 2 visualises how this has modified route modelling.

**Table 6:** Cost values assigned to each vertices

	Main road	Residential road	Footpath	Minor road	Traffic lights	Outlet
To school	0.8	0.5	0.8	1.5	0.5	0.8
Home from school	0.8	0.4	0.8	1.2	0.7	0.5

#### Route to school

Median intersection between GIS-generated routes and GPS routes was greater for the WNR than for the SNR. On average, 53% of WNR intersected with GPS-routes (Table 7) compared with 18% of shortest network routes. One in four WNR completely intersected with the corresponding GPS routes compared with 1.6% of SNR *to school.* For routes less than 3 km long, 28% of WNR intersected completely with the corresponding GPS routes.

**Table 7:** Summary of SNR and WNR overlap with GPS routes for walking routes *to school*

	Mean	SD	Median	Min	Max	Range
GPS route length (km)	1.55	0.88	1.41	0.07	5.18	5.12
SNR length (m)	3.14	1.84	2.79	0.16	14.03	13.86
SNR intersect distance (m)	0.18	0.21	0.11	0	1.65	1.65
SNR intersect with GPS (%)	18	23	8	0	100	100
WNR length (m)	1.68	0.83	1.64	0.15	4.40	4.25
WNR intersect with GPS distance (m)	0.77	0.69	0.57	0.09	3.36	3.35
WNR intersect with GPS (%)	53	39	44	0	100	100

**Fig. 3:**
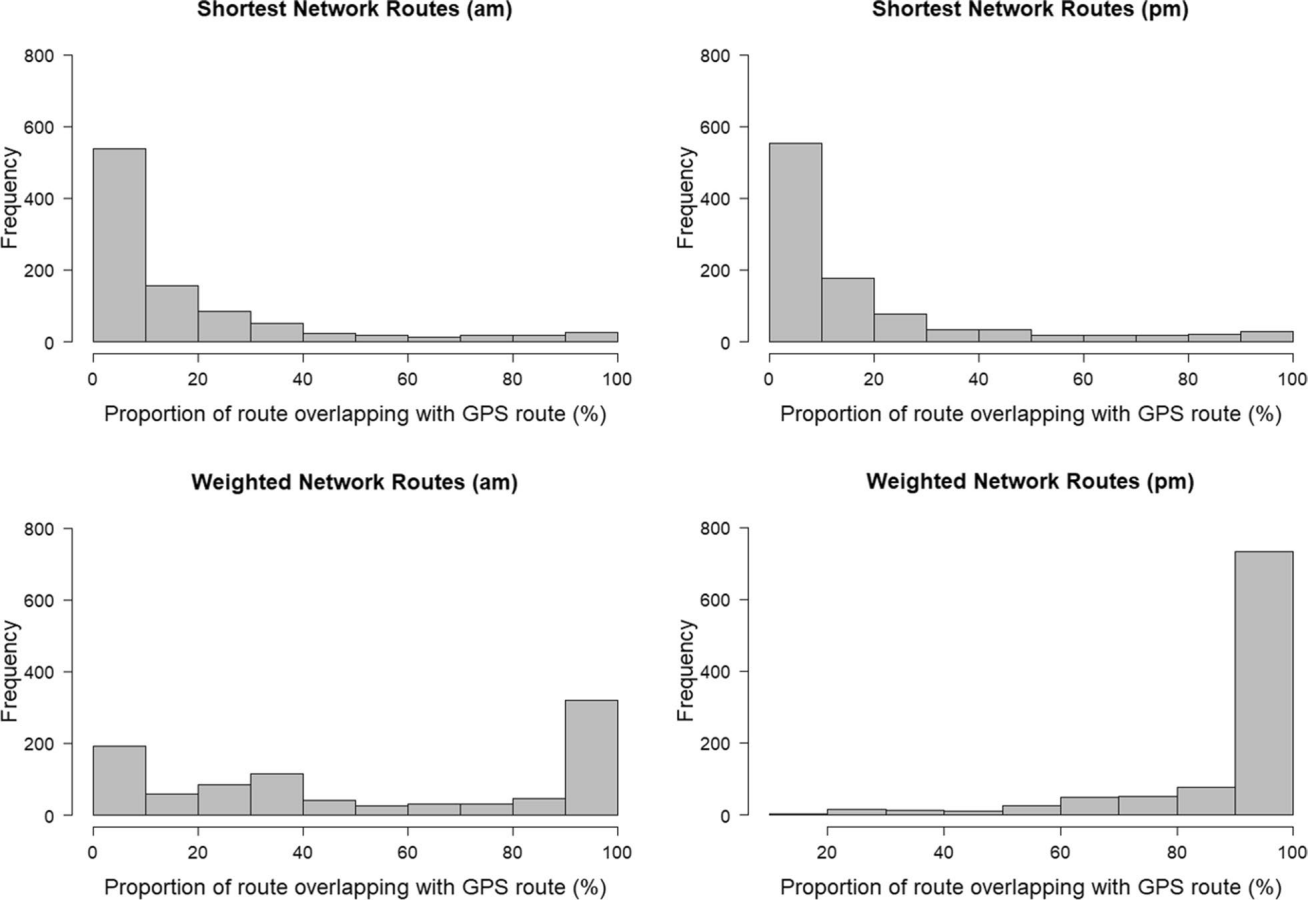
Distribution of intersection between shortest network routes and weighted network routes with GPS route for routes to school (am) and routes home from school (pm)

For SNR, 7% of routes to school intersected completely with GPS routes shorter than 1 km compared with 43% of the WNR *to school*. Route intersection of WNR decreased for route lengths greater than 4 km (n = 10 routes). Distribution of WNR intersection was negatively skewed (skew = 0.02) (Fig. 3) and SNR intersections were positively skewed.

#### Route home from school

Median intersection between the GPS routes and GIS generated routes was 100% for the WNR and 8% for SNR. Mean percentage of intersection decreases as route length increases. More than half of WNRs (56%) completely intersected with the corresponding GPS routes (Table 8) compared with 1.84% of shortest network routes. Median intersection between WNR, SNR and GPS routes was 91% and 18% respectively. For SNR less than 1 km long, 7% routes completely intersected with the GPS recorded routes. The positively skewed distribution for SNR intersections highlighted that the intersection rates were poorer and much less frequent than WNR.

**Table 8:** Summary of SNR and WNR overlap with GPS routes for walking routes *home*

	Mean	SD	Median	Min	Max	Range
GPS route length (m)	1.51	0.91	1.37	0.10	7.17	7.07
SNR length (m)	3.14	1.87	2.80	0.30	14.03	13.73
SNR intersect distance (m)	0.18	0.21	0.10	0	1.65	1.65
SNR intersect with GPS (%)	18	23	8	0	100	100
WNR length (m)	1.71	0.87	1.58	0.15	6.92	6.76
WNR intersect with GPS distance (m)	1.46	0.74	1.40	0.11	4.07	3.96
WNR intersect with GPS (%)	91	17	100	10	100	90

#### Multilevel regression model

The WNE, both along routes to school and along routes home, were positively associated with exposure to the RFE calculated from the GPS routes (p < 0.001). The regression coefficients and standard error (SE) are shown in Table 9.

**Table 9:** Multilevel regression model

Fixed effect	Value	SE	t-value	p-value
Route to school				
Intercept	0.81	1.17	0.48	0.63
WNE^a^	1.42	0.07	19.43	< 0.001
Route home from school				
Intercept	0.66	0.34	1.95	0.05
WNE	1.15	0.04	25.65	< 0.001

^a^Weighted network exposures (WNE) to the RFE defined as the number of food outlets within 100 m of the weighted network route

## Discussion

### Main findings

This paper describes a novel method to model walking routes *to school* and *home* on a large scale with a known accuracy. Our method calculated walking commutes with more than three times greater accuracy than shortest network routes for the route to school and more than 12 times greater accuracy for the route home. We focussed on walking routes as walkers are the most challenging to model and currently, GIS methods used to model walking commutes that contribute to daily mobility measures are inadequate. Our method highlights the accuracy of large-scale GIS-modelled daily mobility measures can be greatly improved by using GPS data to inform GIS methods. It is possible to apply this method to different subgroups of the population to reflect how they may commute and to model different environments that compose daily mobility such as hobbies or weekends.

Analysing GPS-derived walking routes allowed understanding of which built-environment characteristics are associated with route choice along walking commutes *to school* and *home from school*. Measuring built-environment features along walking routes suggests that blue space, traffic light count, food outlets and road type are important built-environment factors in child walking routes *to school* and *home from school*. Our study supports findings that children use crossings with traffic lights when available and avoid walking along main roads [11]. Traffic lights are usually placed for pedestrians to safely cross the road and so children may choose to travel via traffic lights when they are traversing main roads in order to safely cross. Children prefer to traverse residential streets and along footpaths which may explain why accidents were not associated with route choice since accidents tend to occur on busier roads.

The difference between built-environment characteristics along SNR and GPS routes was greatest for the walk *home*. This is consistent with evidence reported elsewhere [41] and highlights that built-environment features have a greater impact on child route choice on the way *home*. On the walk *to school*, there is a time constraint whereas on the route home, without time pressure, children may be more influenced by the built-environment e.g. walking home via the park or a food outlet.

The number of food outlets was significantly less along GPS routes when compared with SNR. This supports the findings by Harrison et al. [8] that SNR overexpose children to the food environment. Furthermore, Dessing et al. [11] and Harrison et al. [8] found that the shortest network distance was a suitable proxy for the distance that a child travels. Our results support this finding for routes *to school* and routes *home*. Children generally traverse a short route on their commute to and from school but their route choice is not necessarily determined by choosing the absolute shortest route. Often, the shortest network route would be along main roads and our results suggest that children avoided walking along the busiest roads.

Woodland was not significantly associated with route choice, either to or from school but previous research suggested children may avoid wooded areas because they are perceived to be unsafe [42, 43]. However, the research that reported children may avoid wooded areas was based on interviews with parents. The lack of significant association suggests that woodland is not an important factor for children deciding which route they chose to take.

Modifying the shortest route calculation by using a weighted network to generate walking routes and the associated exposures greatly improved the accuracy of GIS-modelled routes and exposures. By considering built-environment features that are associated with route choice when modelling walking routes *to school* and *home*, the intersection between modelled routes and GPS routes was greatly improved, particularly for routes *home*. Furthermore, exposure to the food environment was significantly associated with exposures calculated from GPS data for both route *to school* exposures and route *home* exposures. To our knowledge, this has not been documented before. Previous studies have suggested that GPS data should be collected for accurate measures of environmental exposures, but we show that GIS-modelled routes and exposures can be obtained with a known accuracy and used for large-scale studies population level studies instead of small samples of people who have consented to wear devices.

#### Strengths

This study contributes to measuring population-level daily-mobility for individuals by providing a method to generate walking routes to and from school with known accuracy. The method differentiates between the walk *to school* and the walk *home* which, to our knowledge, has not be documented before in GIS-modelled daily mobility measures. The methods presented here can be used to produce dynamic GIS-generated population-level exposures which is important, particularly in public health research, where the results of population-level research contribute to the evidence base that policymakers draw from.

This method has the potential to be adapted for different subgroups of the population and aspects of daily mobility. For example, different modes of transport and calculating exposures to other built-environment measures such as pollution and green space.

#### Limitations

The method presented in this paper calculates one route per child for the walk *to school* and one route for the walk *home*. We assumed that children walked the same route each day and defined a walking routes as directly to school and home. However, children may not walk the same route every day. For example, children may commute via a friend’s home or they may not live in just one home, but two, and so have different start and end locations. However, as this is a population-level model, this is a reasonable assumption to make and the majority of route scenarios have likely been captured.

#### Future work

Further work should explore the cost values of the network and the routing algorithm that is used in the current model. This model uses the Dijkstra’s algorithm and a weighted network to generate routes that represent walking routes to and from school. The Dijkstra algorithm was the only network routing algorithm explored in this investigation. However, other shortest network routing algorithms such as A* algorithm K-shortest path could be used to reduce computing time in larger datasets. Furthermore, travel time should be explored in future work.

Walking routes home were more closely associated with the GPS data than routes to school. The cost values applied to the network were informed by the results of two logistic regression models (route *to school* and route *home*) but further investigation is needed to explore whether the cost values assigned to the walking routes home are more appropriate than the cost assigned for routes to school. The route intersection was far greater between the GPS data and the WNR than the intersection between the GPS data and the shortest network routes to school; and the exposures along the route to school generated from the weighted network were significantly associated with the GPS exposures. However, we did not explore whether the cost values applied to the network are the optimum cost values for modelling children’s walking routes to and from school. Future work could explore: (i) optimum network cost values through the application of more complex machine learning principles (ii) whether factors that influence route choice differ between children who live in urban areas and rural areas.

#### Implications

This study demonstrates that GIS can be used to generate population-level exposures with known accuracy. Generating routes using a shortest network algorithm and a cost weighted street network has produced exposures to food outlets that are significantly associated with exposures calculated from GPS data. These results support the concept that GIS routing models can reliably emulate real life behaviours at a population-level. Large scale, population-level research is an important aspect of the evidence base that policy makers use. Generating children’s exposure to the RFE along their routes to and from school will be a powerful tool for researchers and policy makers as they attempt to combat increasing obesity rates.

This model has been developed using GPS data from a previously funded UK study [28]. The GPS data was representative of a large geographic area that covered urban and rural areas. The model could therefore be applied, to school children around the UK. Children that live in rural areas tend to live further away from the school that they attend and are more likely to drive or use public transport to commute to school. For children who live within 4800 m of the school that they attend, this model is reliable across urban and rural regions in the UK. However, it should be acknowledged that fewer children in rural regions actively travel to school [44]. This supports the need to develop exposure models for other methods of transport and multi-modal transport opportunities. Furthermore, this methodology can be applied elsewhere in the world; by calculating built-environment characteristics that are associated with route choice; use these results to inform the cost values on the network; produce routes from weighted network and associated exposures.

GPS data is often used as the ‘gold standard’ for representing ‘real-life’ behaviour and is a valuable resource for researchers but it is also important to develop alternative GIS methods that can be used for population-scale research because it is not possible to collect GPS data on a large scale. Other GIS generated exposures have previously been modelled and linked with health data for population-level analysis [45, 46] and provide important contextual data to health records. Undertaking large-scale research studies means that generalizable results are added to the evidence base and these can be used to inform policy and population-level interventions. Both of which are vital in driving large scale reductions in the prevalence of health conditions that affect large numbers of people such as obesity [47].

## Conclusions

Numerous studies have investigated factors and behaviours associated with active travel [48–51], but few studies have characterised built-environment features along walking routes *to school* and *home.* Built-environment factors associated with child walking routes to and from school were length, traffic light count, number of food outlets and road type. These characteristics were used to inform costs along a network to predict children walking to and from school using GIS. This work presents a method to model child walking routes to school and home with known accuracy. This is a novel methodology that provides large potential for developing the model to account for other modes of commuting or other aspects of daily mobility for different subgroups of the population. Ultimately, the method presented here could be used in research to provide population-level evidence to advise policy makers and provide evidence to target public health interventions at people who are most likely to achieve active travel to school and work [52].

## Data Availability

The datasets used and/or analysed during the current study were provided by the PEAR research team as a secondary data source. Please communicate with the corresponding author to contact the data providers.

## Abbreviations

GPS: Global Positioning System
GIS: Geographic Information System
PEAR: Physical Environment and Activity Relationships
RFE: Retail food environment
SNR: Shortest network route
WNR: Weighted network route
WNE: Weighted network exposure
